# The Pathophysiology and Pharmacological Treatment of Huntington Disease

**DOI:** 10.3233/BEN-2012-120267

**Published:** 2013

**Authors:** Connie Pidgeon, Hugh Rickards

**Affiliations:** The Michael Trimble Neuropsychiatry Research GroupDepartment of NeuropsychiatryBSMHFT and University of BirminghamBirminghamUK

**Keywords:** Chorea, dopamine, Huntington disease, non-motor symptoms, pharmacotherapy, tetrabenazine

## Abstract

*Introduction:* Huntington disease (HD) is a progressive neurodegenerative condition characterised by motor, cognitive and behavioural dysfunction, and has an autosomal dominant mode of inheritance. As there is currently no treatment to delay progression of the disease, pharmacological intervention is aimed at symptomatic relief.

*Methods:* We set out to assess the current evidence on the pharmacological treatment of motor and non-motor symptoms in HD by carrying out a systematic literature review across five large scientific databases.

*Results:* The search generated 23 original studies meeting our search criteria. Studies on the following drug classes were obtained: dopamine (DA) depleting agents, neuroleptics, anti-glutamatergic agents, acetylcholinesterase inhibitors, GABA agonists, cannabinoids, antidepressants and potential neuroprotective agents. Tetrabenazine (TBZ), a DA depleting agent, was the only pharmacotherapy shown to have a clinically meaningful, statistically significant effect on chorea. The majority of the reviewed studies focussed on the treatment of motor symptoms of HD.

*Discussion:* Overall, the evidence base for the pharmacological management of HD is poor. There is a clear need for future high quality randomised controlled trials on the symptomatic treatment of HD, particularly on the pharmacotherapy of non-motor symptoms of HD.

## 1. Introduction

Huntington's disease (HD) is a progressive neurodegenerative condition which demonstrates an autosomal-dominant mode of inheritance. HD is characterised by motor, cognitive and behavioural dysfunction [1] and has a prevalence of around 4–10 per 100,000 in Western countries [2]. It is caused by an expanded CAG repeat in the Huntingtin gene (HTT) located on the short arm of chromosome 4 at the IT15 locus, which results in an abnormal polyglutamine sequence in the huntingtin protein (Htt) [2–4]. HD occurs when the number of CAG repeats exceeds 36, and repeats of 40 or more results in full penetrance of the disease [4]. The number of CAG repeats is also inversely proportional to the age of onset of symptoms at a population level [6].

The characteristic symptom of HD is chorea (random, dance-like movements) [7]. Other motor symptoms include dystonia, problems initiating voluntary movements, gait disturbances, tics, myoclonus, dysarthria and saccadic eye movements [7–9]. Significant cognitive and behavioural symptoms also occur, such as depression, dementia, psychosis, impaired executive function, apathy, irritability, aggression and personality changes [7,8]. These symptoms often occur in the early stages of the disease, before the onset of motor symptoms [2].

The mean age of symptom onset is 40 years, although symptoms can begin at any age. There is also an early-onset form of HD (juvenile HD or Westphal variant), defined as onset occurring before 20 years, in which rigidity and bradykinesia are predominant motor symptoms [9]. Life expectancy after diagnosis is around 20 years, during which time the patient may become fully dependent [2]. Death usually occurs by secondary causes such as pneumonia [7], and there is also a high incidence of suicide [9,10].

ISSN 0953-4180/13/$27.50 © 2013 – IOS Press and the authors. All rights reserved

Despite the fact that the location of the HTT gene mutation was identified in 1993 [4], the pathological mechanism by which the Htt protein causes neuronal cell dysfunction is still poorly understood. Htt is expressed in all cells, but particularly affects neurons, resulting in the development of toxic function [2]. This protein forms aggregates or inclusion bodies that are distributed throughout neurons and interact with other proteins, leading to neuronal cell dysfunction and eventual death via a variety of possible mechanisms, including caspase activation [11], mitochondrial dysfunction [12], transcriptional dysregulation [13] and increased sensitivity to excitotoxicity [7]. It is likely that more than one of these processes occur simultaneously [14].

The neuropathological changes of HD are selective [9]. Neurons in the striatum (a key component of the basal ganglia) are predominantly affected by this process and this occurs early in the disease course, leading to atrophy of this area of the brain [15]. Increasing numbers of neurons in other areas are affected as the disease progresses, eventually leading to diffuse brain atrophy [2]. The inhibitory medium spiny GABAergic neurons that project from the striatum to the globus pallidus-pars externa (GPe) are most vulnerable and are first affected [7]. This results in dysfunction within the indirect pathway of the basal ganglia which, along with the direct pathway, controls voluntary movement. Specifically, a decrease in the inhibitory neurotransmitter GABA from the striatum to the GPe results in overinhibition of the subthalamic nucleus (STN) which, via output neurons, causes less inhibition of the thalamus, resulting in an increased release of the excitatory neurotransmitter glutamate to the frontal cortex. The clinical correlate of this pathophysiological process is chorea. Dysfunction of both the indirect and direct motor pathways within the basal ganglia leads to the wide range of motor symptoms present in HD [16–18]. Other frontal-subcortical circuits that involve the basal ganglia are also affected as neuronal loss progresses in this area. These pathways control other cognitive, behavioural and emotional functions, therefore playing a causative role in the non-motor symptoms of HD [19].

To date, no neuroprotective, disease-modifying or curative treatment for HD has been developed [1]. Accordingly, pharmacological intervention is aimed at symptomatic relief [17]. The majority of pharmacotherapy used to treat the motor symptoms is aimed at restoring the balance of neurotransmitters that are involved in the pathogenesis of HD, primarily GABA, dopamine (DA) and glutamate [14,20]. These include DA depleting agents such as tetrabenazine (TBZ), DA $D_2$ receptor antagonists (typical and atypical neuroleptics), anti-glutamatergic agents and GABA-agonists. Dopaminergic agents such as Levodopa are sometimes used to treat rigidity and Parkinsonian symptoms, occuring most often in the juvenile form of HD [9]. Drugs used to treat the behavioural and cognitive symptoms include antidepressants, such as selective serotonin reuptake inhibitors (SSRIs), acetylcholinesterase inhibitors (for memory problems), and neuroleptics, which can be used to treat both psychotic and motor symptoms [1].

HD is a devastating, progressive disease with no current disease-modifying treatment available. Consequently, it is crucial that patients receive the most efficacious symptomatic treatment tailored to their individual needs in order to improve their health-related quality of life. This paper aims to review the current evidence on the efficacy of pharmacological treatment for the motor, behavioural and cognitive symptoms of HD.

## 2. Methods

We conducted a systematic literature review according to the methodology suggested by the PRISMA guidelines [22]. Computerised literature searches were performed across the following five databases: MEDLINE, PsycINFO, EMBASE, Thompson Web of Science, and the Cochrane Central Register of Controlled Trials. Due to the broad scope of terminology used to describe the symptoms of HD, two separate groups of search terms were entered into each database. For both searches the terms 'Huntington*', 'drug', 'medication', and 'pharmaco*' were used. The following search terms were added to the first search to encompass the pharmacotherapy for the motor symptoms: 'chorea', 'motor', 'movement', 'dystoni*' and 'tic'. The search terms 'behav*', 'mood', 'affect', 'psychoses', 'delusion', 'cogniti*', 'depression' and 'anxi*' were added to the second search to find trials on the treatment of the behavioural (psychiatric) and cognitive (neuropsychological) symptoms. In addition, PsycINFO and EMBASE thesauri and the Medical Subject Heading (MeSH) database of controlled vocabulary were used to index papers and find alternative search terms. Moreover, to identify articles that did not come up in the initial searches, the contents tables of journals that publish articles relevant to this topic were reviewed. These included: Brain; The Journal of Neurology, Neurosurgery and Psychiatry; Movement Disorders; Neurology; and Clinical Neuropharmacology. The reference lists of recent review articles were scanned for further relevant studies, and additional searches were carried out in the 'Google Scholar' search engine. Finally, advice was sought from HD experts to ensure any pertinent articles were not overlooked.

The preliminary search generated over 2500 results and was subsequently limited to English language papers published after 1980. Further exclusion criteria included case series, case reports, editorials, commentaries, and letters to the editors of scientific journals. Following removal of unrelated and duplicate papers, over 150 results were still eligible for inclusion. Therefore it was decided to exclude open-label and retrospective trials, and to focus this systematic literature review on double-blind, randomised controlled trials (RCTs) with 20 or more patients, thereby only including RCTs with high quality evidence. As this review is on symptomatic treatment, studies on potential neuroprotective treatments were only included if the main form of assessment was the effect of the drug on HD symptoms.

## 3. Results

Table 1 displays the 23 RCTs on the pharmacological treatment of HD that matched the criteria set out in the Methods section. All but one study [23] were placebo-controlled, further adding to the overall high-quality nature of the studies. The study results, organised by drug class, are discussed in the following paragraphs.

### *3.1. DA depleting agents*

Two RCTs found TBZ to have a positive effect on reducing chorea. A multicentre study by the HSG [24] randomised patients to receive placebo ($n = 30$) or TBZ ($n = 54$) for 12 weeks. The TBZ dose started at 12.5 mg/day and was titrated up to a maximum of 100 mg/day. TBZ treatment resulted in a decline of 5.0 units of chorea severity assessed by the UHDRS, compared to a decline of 1.5 units in the placebo group. A study by Frank et al. [22] randomised 30 patients who had been taking TBZ for at least 2 months into 3 groups, and who then withdrew from TBZ in a staggered fashion over a 5-day period. The efficacy and short half-life of TBZ was demonstrated by a worsening of chorea in the withdrawal and partial withdrawal groups compared to the non-withdrawal group.

### *3.2. Neuroleptics*

Both typical and atypical neuroleptics can be used for the treatment of chorea and psychosis in HD [20]. Both are DA $D_2$ receptor antagonists, however the newer atypical antipsychotics are preferred because they cause less extrapyramidal side-effects [1]. Three RCTs on atypical neuroleptics were found which met the inclusion criteria of the present systematic literature review. Clozapine was studied by van Vugt et al in a RCT with 33 patients [25], and was found to have little beneficial effect on chorea. However, a high proportion of patients experienced adverse effects, meaning that the maximum dose of 150 mg/day was not reached (which could potentially have resulted in a greater efficacy). Tiapride (dose 3g/day), a substituted benzamide, was found by Deroover et al. [25] to have a significant positive effect on chorea in a placebo-controlled crossover study of 29 patients. Conversely, a 2-week study by Roos et al. [26] in which patients took 100 mg/day of tiapride found no significant effects on chorea. However, the lower dose and difference in assessment and rating scales could have contributed to the different outcomes. A recent study by Lundin et al. [27] found pridopidine, a dopaminergic stabiliser, to have a similar effectiveness to placebo.

### *3.3. Anti-glutamatergic agents*

Two similar RCTs were found on amantadine (a NMDA-receptor antagonist). Both trials were of 4-weeks duration and involved 24 patients and similar doses. However, these studies have conflicting results. Verhagen-Metman et al. [28] found 400 mg/day to have a positive effect on chorea and few adverse effects. Conversely, O'Suilleabhain and Dewey [29] showed that amantadine 300 mg/day had no impact on chorea compared to placebo, and more adverse effects were reported. However, 19 patients reported subjective improvement after the treatment phase, compared to 6 after the placebo phase.

Remacemide is a non-competitive NMDA-receptor antagonist. In a RCT by Kieburtz et al. [30] patients receiving remacemide 200 mg/day dose showed an overall improvement in chorea level. A larger multicentre study involving 347 patients by the HSG [31] looked into the effects of remacemide and coenzyme $Q_{10}$ (a neuroprotective agent) on functional decline in early HD. Neither medication was found to have a statistically significant effect, as measured primarily by the change in Total Functional Capacity (TFC). However,

Table 1

Randomised controlled trials on the pharmacological treatment of the motor and non-motor symptoms of Huntington disease

| Study | Country | Treatment duration | Study design | n | Medication | Mechanism of action | Primary outcome measure(s) | Overall efficacy (Compared to placebo) | Concomitant HD medication |
|---|---|---|---|---|---|---|---|---|---|
| Frank et al. (2008) [23]* | USA | 5 days | Parallel | 30 | TBZ | Dopamine depleting agent | UHDRS | Good | Not controlled |
| HSG (2006) [24] | USA | 12 weeks | Parallel | 84 | TBZ | Dopamine depleting agent | UHDRS | Good | Controlled and permitted medication was kept constant |
| van Vugt et al. (1997) [25] | Holland | 31 days | Parallel | 33 | Clozapine | $D_2$ receptor antagonist | UHDRS, AIMS | Poor | Kept constant |
| Deroover et al. (1984) [26] | Belgium | 9 weeks | Cross-over | 29 | Tiapride | $D_2$ receptor antagonist | Chorea-scale and motor activity-scale, subjective measurements of anxiety | Good | Controlled – only anxiolytics and antidepressants allowed |
| Roos et al. (1982) [27] | Denmark | 4 weeks | Cross-over | 22 | Tiapride | $D_2$ receptor antagonist | chorea scale (+ subjective) | None | Kept constant |
| Lundin et al. (2010) [28] | Sweden and Norway | 4 weeks | Parallel | 58 | Pridopidine (ACR16) | Dopaminergic stabiliser | Weighted cognitive score | None | Kept constant |
| Verhagen-Metman et al. (2002) [29] | USA | 4 weeks | Cross-over | 24 | Amantadine | NMDA-receptor antagonist | UHDRS | Good | Not allowed |
| O'Suilleabhain and Dewey (2003) [30] | USA | 2 weeks | Cross-over | 24 | Amantadine | NMDA-receptor antagonist | Chorea-scale (video) | None | Kept constant |
| Kieburtz et al. (1996) [31] | USA | 5 weeks | Parallel | 31 | Remacemide | NMDA-receptor antagonist | HDFCS, HDMRS and TFC | Poor | Controlled and kept constant |
| HSG (2001) [32] | USA | 30 months | Parallel | 347 | Remacemide<br>Coenzyme $Q_{10}$ | NMDA-receptor antagonist<br>Antioxidant | TFC | Poor<br>Poor | Not controlled (other than excluded if taken Coenzyme Q10 in preceding 3 months) |
| Landwehrmeyer et al. (2007) [33] | 8 European Countries | 3 years | Parallel | 537 | Riluzole | Inhibits glutamate release | UHDRS | None | Not allowed |
| HSG (2003) [34] | USA and Canada | 8 weeks | Parallel | 63 | Riluzole | Inhibits glutamate release | UHDRS | Good | Kept constant |
| Kremer et al. (1999) [35] | Canada | 30 months | Parallel | 64 | Lamotrigine | Inhibits glutamate release | TFC, QNE | Poor | Controlled |
| Cubo et al. (2006) [36] | USA and Spain | 12 weeks | Parallel | 30 | Donepezil | Acetylcholin ersterare-inhibitor | UHDRS, ADAS | None | Controlled |
| Shoulson et al. (1989) [37] | USA | 42 months | Parallel | 60 | Baclofen | GABA agonist | TFC | None | Not controlled |
| Curtis et al. (2009) [38] | UK | 15 weeks | Cross-over | 44 | Nabilone | Cannabinoid CB1 and CB2 receptor agonist | UHDRS | Poor | Kept constant |

Table 1, continued

| Study | Country | Treatment duration | Study design | n | Medication | Mechanism of action | Primary outcome measure(s) | Overall efficacy (Compared to placebo) | Concomitant HD medication |
|---|---|---|---|---|---|---|---|---|---|
| Como et al. (1997) [39] | USA | 4 months | Parallel | 30 | Fluoxetine | Selective serotonin re-uptake inhibitor | TFC | None | Controlled – antidepressants not allowed, others kept constant |
| Puri et al. (2005) [40] | UK, USA, Canada, Australia | 1 year | Parallel | 135 | Ethyl-EPA | Various – targets mitochondrial function | UHDRS | None | Not controlled (but changes were noted) |
| HSG (2008) [41] | USA and Canada | 6 months | Parallel | 316 | Ethyl-EPA | Various – targets mitochondrial function | UHDRS | None | Controlled |
| Beglinger et al. (2009) [42] | USA | 10 weeks | Cross-over | 20 | Atomoxetine | Noradrenaline reuptake inhibitor | Subjective (self-reported) | None | Kept constant |
| Verbessem et al. (2003) [43] | Belgium | 1 year | Parallel | 41 | Creatine | Regulates energy metabolism | UHDRS | None | Kept constant |
| Blackwell et al. (2008) [44] | UK | 3 weeks | Cross-over | 20 | Modafinil | Various – analeptic | Neuropsychological tests | Poor | Not controlled |
| Kieburtz et al. (2010) [45] | UK and USA | 3 months | Parallel | 91 | Latrepirdine | Antihistamine – stabilises mitochondrial membranes | UHDRS and MMSE | Poor | Not controlled |

Abbreviations: n = number of patients; HSG = The Huntington Study Group; Ethyl-EPA = ethyl-eicosapentaenoic acid; UHDRS = Unified Huntington's Disease Rating Scale; AIMS = Abnormal Involuntary Movement Scale; TFC = Total Functional Capacity Score; HDMRS = Huntington's Disease Motor Rating Scale; MMSE = Mini Mental State Examination; QNE = Quantified Neurological Examination; ADAS = Alzheimer's Disease Assessment Scale; subjective = non-validated rating scale; video = video-blinded rating; *Not placebo-controlled.

the results may have been influenced by concomitant HD medication, as this was not controlled.

Riluzole is a glutamate release inhibitor. Two double-blind, placebo-controlled RCTs were found that investigate its effect on chorea in HD. In a large study by Landwehrmeyer et al. [32], 537 patients were randomised to receive riluzole 50 mg/day or placebo in a 2:1 format over 3 years. Use of other antichoreic medication was not permitted, adding to the study's reliability. Whilst only 71% of patients completed the trial, the main reason for this was withdrawal due to concomitant medication, rather than adverse effects. No beneficial effects on chorea were found. The dosage effects of riluzole were investigated in a smaller study of 63 patients by the HSG [33], with contrasting results to the previous study. After 8 weeks of treatment, riluzole 200 mg/day reduced chorea compared to placebo. Finally, Kremer et al. [34] conducted a RCT of lamotrigine, which showed improvement of chorea in the active arm and a patient-reported improvement in symptoms, although these were not statistically significant.

### 3.4. *Acetylcholinesterase inhibitors*

Acetylcholinesterase inhibitors are used to treat cognitive decline, a common symptom of HD. A placebo-controlled trial of 30 patients by Cubo et al. [35] found that donepezil was ineffective in improving either motor or cognitive dysfunction in HD.

### 3.5. *GABA agonists*

Baclofen was studied in a RCT involving 60 patients by Shoulson et al. [36], showing no benefit over placebo.

### 3.6. *Cannabinoids*

One pilot RCT by Curtis et al. [37] focused on nabilone, a cannabinoid agent that is agonistic at both CB1 and CB2 receptors. In this cross-over pilot trial, 44 patients were randomised to receive nabilone 1 or 2 mg, or placebo over a period of 15 weeks. There was no improvement in the nabilone arm on the primary outcome measure (total motor score on the UHDRS). However, there was some improvement in the secondary neuropsychiatric outcome measures, such as irritability? (chorea and irritability I think).

### 3.7. *Antidepressants*

In a 4 month study by Como et al. [38], 30 non-depressed HD patients were randomised to either fluoxetine or placebo to test if fluoxetine has any clinical benefit for non-depressed patients. Outcome measures included change in TFC, as well as in other rating scales of cognitive, behavioural and neurological changes. Fluoxetine was not found to significantly improve these symptoms, although there was a small reduction in agitation in the treatment arm. No RCTs were found on the treatment of depression in HD patients.

### 3.8. *Potential neuroprotective agents*

Two large, multicentre RCTs were found on ethyl-EPA. Both trials (Puri et al. [39] and HSG [40]) found that ethyl-EPA did not have a greater efficacy than placebo.

Other neuroprotective agents that have been tested in RCTs include atomoxetine (Beglinger et al. [41]), creatine (Verbessem et al. [42]), and modafinil (Blackwell et al. [43]) for cognitive dysfunction, and latrepirdine (Kieburtz et al. [44]) for cognitive, behavioural and motor symptoms. No beneficial effects of atomoxetine or creatine were reported, and modafinil and latrepirdine had only minor positive effects.

## 4. Discussion

This paper systematically reviewed the current literature on the symptomatic treatment of HD. Searches across 5 databases generated over 2500 results. The end result after the limits and exclusion criteria were applied was 23 double-blind RCTs, with all but one [23] being placebo-controlled. Due to the strict exclusion criteria, the studies included in this paper were of the highest quality evidence available on this topic. The main result of this paper is that, out of the 23 RCTs, the DA-depleting agent TBZ is the pharmacological agent with the greatest evidence base for improving the motor symptoms of HD.

TBZ was the only pharmacotherapy shown to have a clinically meaningful, statistically significant benefit on chorea. However, in the HSG study [24] serious adverse events occurred exclusively in the active arm, and these patients had worse scores in functional outcome. In clinical situations, these factors should be weighed up against its benefit in reducing chorea. Neuroleptics are used clinically to treat both chorea and psychoses, but the three studies on neuroleptics [25–27] showed no conclusive evidence for any beneficial effect on chorea. Also, none of these three studies looked into the effect on psychoses. The drug class with the greatest number of RCTs found was anti-glutaminergic agents. Two studies on amantadine [29,30] had conflicting results in terms of the effect on chorea; this difference in outcome could be due to the relatively small sample sizes, or differences in baseline characteristics. For example, there was a considerable difference in average age between the two cohorts. Remacemide [31] was shown to have some beneficial effect on chorea in one RCT. The efficacy of riluzole on reducing chorea was demonstrated at a dose of 200 mg/day, however this was associated with a considerable level of hepatic toxicity [34], suggesting it should not be used clinically at this dose. Nabilone [38] was found to have a beneficial impact on neuropsychiatric symptoms, an important finding considering the high prevalence and severity of these symptoms in HD. No other medications had any significant positive effect on the symptoms of HD.

The difference in number between the 2500 initial search results and the 23 RCTs that were included in this review shows that the symptomatic treatment of HD is based overall on low-quality evidence from different types of studies, such as case reports, case series and open-label studies. Some drugs, for example levodopa, were not included in this review as there were no trials that matched the search criteria. This illustrates the scarcity of high-quality evidence for some HD medications. The reason that there are few high-quality trials may be related to the fact that HD is a rare condition, making it difficult to recruit enough patients to achieve high statistical power. Also, the nature of HD symptoms may increase the likelihood of patients dropping out of studies, and may affect compliance with study protocols.

The majority of the reviewed studies were on motor function in HD, perhaps because changes in these symptoms are easier to measure objectively compared to assessing changes in behavioural and cognitive symptoms. This is an important finding, as it is often non-motor symptoms that have the greatest impact on a patient's health-related quality of life [46]. Furthermore, suicide is common in HD [1,8,9]. Hence, it is crucial that any behavioural symptoms that might make suicide more likely are treated as effectively and efficiently as possible.

The majority of trials used objective measurements to determine the effect of symptomatic treatment. For example, 9 studies used changes to the UHDRS as their primary outcome measure. This is usually perceived to be beneficial, as it increases the overall reliability and consistency of the results. However, perhaps subjective measurements such as patient-reported scales of well-being are more important than clinician-judged outcomes. To illustrate this, previous studies have suggested that objective improvement in chorea may not correlate with an improvement in a patient's mood or function [34].

Although this review only included trials with the highest level of evidence available on this topic, there are still several shortcomings which need to be addressed. Firstly, over 50% of the studies had a fairly small sample size ($n < 50$). Secondly, the use of concomitant medication was not controlled in 6 of the studies, which could have had a significant effect on treatment outcome. In addition, not all studies stated the baseline characteristics of the patients before starting treatment, which may have meant the two arms were different from the start. In two studies the patients were recruited by advertisements, and in several studies the patients were recruited from specialist clinics, both of which will have led to a degree of sampling bias. In some studies a substantial number of patients were lost to follow up, possibly leading to further bias. Furthermore, there was a large variation in study duration, and medications may not have had an effect within the short duration of some studies. A particular difficulty in interpreting results is the difference in outcome measures, as some studies used standardised methods such as the UHDRS and were therefore easy to compare, whereas others used self-report or subjective rating scales.

There is a clear need for more high quality, placebo-controlled, double-blind RCTs in the future on the symptomatic treatment of HD. Further trials are needed with a large number of patients (ideally $n > 100$ to ensure adequate statistical power), and with a prolonged duration to ensure that the treatment effects are not overlooked. It is also important to ensure that concomitant HD medication is controlled, and that outcome measures are sensitive and comparable across studies. Additionally, it is vital that more RCTs are carried out on the treatment of non-motor symptoms of HD, of which there is a clear deficiency in the literature.

## 5. Conclusion

The aim of this paper was to systematically review the available literature on the pharmacological treatment of symptoms in HD. Our extensive literature search and strict exclusion criteria meant that only high quality RCTs were included. Despite this, the evidence base for the pharmacological management of HD

is poor. Only one medication, TBZ, was consistently shown to have significant efficacy in the treatment of chorea. The evidence for the treatment of behavioural and cognitive symptoms was especially poor, emphasised by the low number of RCTs on these symptoms. Based on these results, no definite treatment recommendations can be made. The results suggest that symptomatic intervention in HD is based more on the experience and knowledge of the clinician, as opposed to rigorous evidence provided by the literature [46]. As patients with HD are treated with medications that have very little evidence for their effectiveness, the decision to treat patients with pharmacotherapy should take into consideration multiple factors. Clinicians working with patients with HD should always keep in mind the importance of supportive care and management by the multi-disciplinary team when considering treatment options.

## References

[1] O.R. Adam and J. Jankovic, Symptomatic treatment of Huntington disease, *Neurotherapeutics* **5** (2008), 181–197.

[2] C.A. Ross and S.J. Tabrizi, Huntington's disease: from molecular pathogenesis to clinical treatment, *Lancet Neurol* **10** (2011), 83–98.

[3] J.F. Gusella, N.S. Wexler, P.M. Conneally, S.L. Naylor, M.A. Anderson, R.E. Tanzi et al., A polymporphic DNA marker genetically linker to Huntington's disease, *Nature* **306** (1983), 234–238.

[4] The Huntington's Disease Collaborative Research Group. A novel gene containing a trinucleotide repeat that is expanded and unstable on Huntington's disease chromosomes, *Cell* **72** (1993), 971–983.

[5] W. Philips, K.M. Shannon and R.A. Barker, The current clinical management of Huntington's disease, *Mov Disord* **23** (2008), 1491–1504.

[6] S.J. Tabrizi, D.R. Langbehn, B.R. Leavitt, R.A.C. Roos, A. Durr, D. Crauford et al., Biological and clinical manifestations of Huntington's disease in the longitudinal TRACK-HD study: cross sectional analysis of baseline data, *Lancet Neurol* **8** (2009), 791–801.

[7] C. Wider and R. Luthi-Carter, Huntington's disease: clinical and aetiological aspects, *Schweiz Arch Neurol Psychiatr* **157** (2006), 378–383.

[8] B.A. Haskins and M.B. Harrison, Huntington's disease, *Curr Treat Option N* **2** (2000), 243–262.

[9] F.O. Walker, Huntington's disease, *Lancet* **369** (2007), 218–228.

[10] D. Paleacu, Tetrabenazine in the treatment of Huntington's disease, *Neuropsychiatr Dis Treat* **3** (2007), 545–551.

[11] S. Krobitsch and A.G. Kazantsev, Huntington's disease: from molecular basis to therapeutic advances, *Int J Biochem Cell B* **43** (2010), 20–24.

[12] J.T. Greenamyre, Huntington's disease – making connections, *N Engl J Med* **365** (2007), 518–529.

[13] E. Roze, F. Saudou and J. Caboche, Pathophysiology of Huntington's disease: from huntingtin functions to potential treatment, *Curr Opin Neurol* **21** (2008), 497–503.

[14] S. Frank, Tetrabenazine: the first approved drug for the treatment if chorea in US patients with Huntington disease, *Neuropsychiatr Dis Treat* **6** (2010), 657–665.

[15] H. Ryu, H.D. Rosas, S.M. Hersch and R.J. Ferrante, The therapeutic role of creatine in Huntington's disease, *Pharmacol Therapeut* **108** (2005), 193–207.

[16] S. Tekin and J.L. Cummings, Frontal-subcortical neuronal circuits and clinical neuropsychiatry: an update, *J Psychosom Res* **53** (2002), 647–654.

[17] J.P.P. van Vugt and R.A.C. Roos, Huntington's disease: options for controlling symptoms, *CNS Drugs* **11** (1999), 105–123.

[18] V.M. Andre, C. Cepeda and M. Levine, Dopamine and glutamate in Huntington's disease: a balancing act, *CNS Neurosci Ther* **16** (2010), 163–178.

[19] R.M. Bonelli and J.L. Cummings, Frontal-subcortical circuitry and behaviour, *Dialogues Clin Neurosci* **9** (2007), 141–151.

[20] R. Bonelli and P. Hofmann, A systematic review of the treatment studies in Huntington's disease since 1990, *Expert Opin Pharmacother* **7** (2007), 1–13.

[21] D. Moher, A. Liberati, J. Tetzlaff and D.G. Altman, Preferred reporting items for systematic reviews and meta-analyses: the PRISMA statement, *Ann Intern Med* **151** (2009), 264–269.

[22] S. Frank, W. Ondo, S. Fabn, C. Hunter, D. Oakes, S. Plumb et al., A study of chorea after tetrabenazine withdrawal in patients with Huntington's disease, *Clin Neuropharmacol* **31** (2008), 127–133.

[23] Huntington Study Group, Tetrabenazine as antichorea therapy in Huntington disease: a randomised control trial, *Neurology* **66** (2006), 366–372.

[24] J.P.P. van Vugt, S. Siesling, M. Vergeer, E.A. van der Velde and R.A.C. Roos, Clozapine versus placebo in Huntington's disease: a double blind randomised comparative study, *J Neurol Neurosurg Psychiatry* **63** (1997), 35–39.

[25] J. Deroover, F. Baro, R.P. Bourguignon and P. Smets, Tiapride versus placebo: a double-blind comparative study in the management of Huntington's chorea, *Curr Med Res Opin* **9** (1984), 329–338.

[26] R.A.C. Roos, O.J.S. Buruma, G.W. Bruyn, B. Kemp and E.A. van de Velde, Tiapride in the treatment of Huntington's chorea, *Acta Neurol Scand* **65** (1982), 45–50.

[27] A. Lundin, E. Dietrichs, S. Haghighi, M.L. Goller, A. Heiberg, G. Loutfi et al., Efficacy and safety of the dopaminergic stabilizer pridopidine (ACR16) in patients with Huntington's disease, *Clin Neuropharm* **33** (2010), 260–264.

[28] L. Verhagen-Metman, M.J. Morris, C. Farmer, N.P. Gillespie, K. Mosby, J. Wuu et al., Huntington's disease: a randomised, controlled trial using the NMDA-antagonist amantadine, *Neurology* **59** (2002), 694–699.

[29] P. O'Suilleabhain and R.B. Dewey, A randomised trial of amantadine in Huntington disease, *Arch Neurol* **60** (2003), 996–998.

[30] K. Kieburtz, A. Feigin, M. McDermott, P. Como, D. Adwender, C. Zimmerman et al., A controlled trial of remacemide hydrochloride in Huntington's disease, *Mov Disord* **11** (1996), 273–277.

[31] Huntington Study Group, A randomized, placebo-controlled trial of coenzyme $Q^{10}$ and remacemide in Huntington's disease, *Neurology* **57** (2001), 397–404.

[32] G.B. Landwehrmeyer, B. Dubois, J.G. de Yebenes, B. Kremer, W. Gaus, P.H. Kraus et al., Riluzole in Huntington's disease: a 3-year, randomised controlled study, *Ann Neurol* **62** (2007), 262–272.

[33] Huntington Study Group, Dosage effects if riluzole in Huntington's disease: a multicentre placebo-controlled study, *Neurology* **61** (2003), 1551–1556.

[34] B. Kremer, C.M. Clark, E.W. Almqvist, L.A. Raymond, P. Graf, C. Jacova et al., Influence of lamotrigine on progression of early Huntington's disease: a randomized clinical trial, *Neurology* **53** (1999), 1000–1011.

[35] E. Cubo, K.M. Shannon, D. Tracy, J.A. Jaglin, B.A. Bernard, J. Wuu et al., Effect of donepezil on motor and cognitive function in Huntington disease, *Neurology* **67** (2006), 1268–1271.

[36] I. Shoulson, C. Odoroff, D. Oakes, J. Behr, D. Goldblatt, E. Caine et al., A controlled clinical trial of baclofen as protective therapy in early Huntington's disease, *Ann Neurol* **25** (1989), 252–259.

[37] A. Curtis, I. Mitchell, S. Patel, N. Ives and H. Rickards, A pilot study using nabilone for symptomatic treatment in Huntington's disease, *Mov Disord* **24** (2009), 2254–2259.

[38] P.G. Como, A.J. Rubin, C.F. O'Brien, K. Lawler, C. Hickey, A.E. Rubin et al., A controlled trial of fluoxetine in nondepressed patients with Huntington's disease, *Mov Disord* **12** (1997), 397–401.

[39] B.K. Puri, B.R. Leavitt, M.R. Hayden, C.A. Ross, A. Rosenblatt, J.T. Greenamyre et al., Ethyl-EPA in Huntington's disease: a double-blind, randomized, placebo-controlled trial, *Neurology* **65** (2005), 286–292.

[40] Huntington Study Group TREND-HD Investigators. Randomized controlled trial of ethyl-eicosapentaenoic acid in Huntington disease, *Arch Neurol* **65** (2008), 1582–1589.

[41] L.J. Beglinger, W.H. Adams, H. Paulson, J.G. Fiedorowicz, D.R. Langbehn, K. Duff et al., Randomised controlled trial of atomoxetine for cognitive dysfunction in early Huntington disease, *J Clin Psychopharmacol* **29** (2009), 484–487.

[42] P. Verbessem, J. Lemiere, B.O. Eijnde, S. Swinnen, L. Vanhees, M. van Leemputte et al., Creatine supplementation in Huntington's disease: a placebo-controlled pilot trial, *Neurology* **61** (2003), 925–930.

[43] A.D. Blackwell, N.S. Paterson, R.A. Barker, T.W. Robbins and B.J. Sahakian, The effects of modafinil on mood and cognition in Huntington's disease, *Psychopharmacology* **199** (2008), 29–36.

[44] K. Kieburtz, M.P. McDermott, T.S. Voss, J. Corey-Bloom, L.M. Deuel, R. Dorsey et al., A randomized, placebo-controlled trial of latrepirdine in Huntington disease, *Arch Neurol* **67** (2010), 154–160.

[45] E. van Duijn, Treatment of irritability in Huntington's disease, *Curr Treat Option Neurol* **12** (2010), 424–433.

[46] R.M. Bonelli and G.K. Wenning, Pharmacological management of Huntington's disease: and evidence-based review, *Curr Pharm Des* **12** (2006), 2701–2720.
